# Alleviating heat stress effects in poultry: updates on methods and mechanisms of actions

**DOI:** 10.3389/fvets.2023.1255520

**Published:** 2023-09-27

**Authors:** Okanlawon M. Onagbesan, Victoria Anthony Uyanga, Oluwadamilola Oso, Kokou Tona, Oyegunle Emmanuel Oke

**Affiliations:** ^1^Department of Animal Physiology, Federal University of Agriculture, Abeokuta, Nigeria; ^2^Department of Animal Science, Iowa State University, Ames, IA, United States; ^3^Centre of Excellence in Avian Sciences, University of Lome, Lomé, Togo

**Keywords:** heat stress, poultry, nutrition, management, environment, welfare

## Abstract

Heat stress is a threat that can lead to significant financial losses in the production of poultry in the world’s tropical and arid regions. The degree of heat stress (mild, moderate, severe) experienced by poultry depends mainly on thermal radiation, humidity, the animal’s thermoregulatory ability, metabolic rate, age, intensity, and duration of the heat stress. Contemporary commercial broiler chickens have a rapid metabolism, which makes them produce higher heat and be prone to heat stress. The negative effect of heat stress on poultry birds’ physiology, health, production, welfare, and behaviors are reviewed in detail in this work. The appropriate mitigation strategies for heat stress in poultry are equally explored in this review. Interestingly, each of these strategies finds its applicability at different stages of a poultry’s lifecycle. For instance, gene mapping prior to breeding and genetic selection during breeding are promising tools for developing heat-resistant breeds. Thermal conditioning during embryonic development or early life enhances the ability of birds to tolerate heat during their adult life. Nutritional management such as dietary manipulations, nighttime feeding, and wet feeding often, applied with timely and effective correction of environmental conditions have been proven to ameliorate the effect of heat stress in chicks and adult birds. As long as the climatic crises persist, heat stress may continue to require considerable attention; thus, it is imperative to explore the current happenings and pay attention to the future trajectory of heat stress effects on poultry production.

## Introduction

Every animal production system experiences stress because of the numerous stressors on the farms. Stress is a biologically adaptive response to re-establish homeostasis (1). Heat stress is a variant of environmental stress caused by an increase in environmental temperature (and humidity) beyond the thermotolerance of an animal. Poultry birds possess a narrow range of thermoregulatory thresholds and are sensitive to environmental temperatures, which can pose as a stressor. Over the years, genetic selection has brought about rapid improvement in poultry birds’ growth and muscle development, but not in the physiological enhancement of the thermoregulatory system. Heat stress is a threat to the environment that can result in significant financial losses in the production of poultry in the world’s tropical and arid regions. High environmental temperature, airspeed, radiant heat, and humidity interact to cause heat stress; of these, high ambient temperature has a significant impact (2–4). Heat stress is a physiological result of the imbalance between heat energy production and heat energy flow from the animal to the environment (5). The degree of heat stress (mild, moderate, severe) experienced by the animal depends on a combination of different factors but is not limited to the environmental factors (humidity, thermal irradiation), individuality (animal’s thermoregulatory ability, metabolic rate, age) and characteristics of the heat stress (intensity and duration).

The adaptive response of poultry to heat stress conditions is intricate and complex (1). When heat stress sets in, poultry birds respond physiologically and behaviourally in an attempt to seek homeostasis and restore comfort (6). Poultry birds often attempt to increase latent heat loss through increased respiratory rate, but these mechanisms seem insufficient, and additional intervention is required. These significant physiologic, neuroendocrine, and behavioral alterations result in elevated mortality, decreased feed intake, decreased final body weight, decreased meat and egg quality, and elevated feed conversion ratios in poultry. In light of climate change and its associated financial losses, thermal stress has thus been of utmost concern to poultry producers, and various approaches have been used to address this issue (7). Literature has highlighted different amelioration and mitigation strategies adopted for poultry production and this review seeks to synchronize these findings, examine their shortfalls, and make recommendations for improvement.

### Impacts of heat stress on poultry production

The negative effect of heat stress has been reported on the physiology, health, production, welfare and behaviors of poultry birds. In heat-stressed chickens, oxidative stress, acid–base imbalance, and suppressed immunity are some of the physiological changes accompanying heat stress. Oxidative stress is frequently caused by an imbalance between the generation of ROS and the efficacy of the antioxidant defence system (8). Numerous cell components, including lipids, proteins, and DNA, are susceptible to permanent damage from the free radical generation that frequently follows oxidative stress.

An animal’s physiological response to stress involves a variety of systems such as the neuroendocrine, immune system, circulatory and digestive systems. For instance, heat stress can impair the feeding process, nutrient absorption and utilization although water intake increases rapidly. Richards et al. (9) and Morera et al. (10) highlighted the mechanism involved in the diminished voluntary feed intake. The authors pointed out that the upregulation of adipokines secretion (leptin and adiponectin) and the expression of their receptors can negatively regulate feed intake and calorie consumption thus resulting in decreased metabolic heat production. This mechanism may result in short-term heat balance but eventually affect the functionality of the digestive system due to changes in motility and flux patterns, secretory activity, content viscosity, and pH (11, 12). The decline in trypsin, chymotrypsin and amylase (intestinal secretion) due to reduced feed intake often results in impairment of digestive functionality, nutrient digestibility, and rapid feed transit (11, 12).

Under heat stress conditions, hypoperfusion and an increase in blood flow to the skin surface occur as an adaptive response of the circulatory system to stabilize blood pressure and promote heat loss (13). Hypoperfusion which can lead to a reduction in oxygen availability in organs when combined with intestinal changes often results in oxidative stress and inflammation.

Immunosuppressing effect of thermal challenge has been documented and this demonstrates that the neuroendocrine system plays an important role in the normal physiological functioning and homeostasis of birds during thermal challenge. The sympathoadrenal medullary axis is activated and controls homeostasis during the early phases of thermal challenge. A hormonal increase of catecholamines (epinephrine and norepinephrine) and glucocorticoids occurs in the adrenal medulla as a result of the impulse transmitted by the sympathetic nerves as a result of elevated temperature. Due to this, a decrease in muscle and liver glycogen and an increase in respiratory rate are experienced (14). The hypothalamic–pituitary–adrenal axis is stimulated as the length of the stress period increases. Corticotrophin-releasing hormone, which is secreted in reaction to stress, causes the pituitary to release a hormone called adrenocorticotrophic hormone (ACTH). The release and synthesis of corticosteroids by the adrenal glands are increased by ACTH (15). The increase in corticosterone is often accompanied by an increase in heterophiles often resulting in increased heterophile: lymphocyte (16, 17). Reduced number of lymphocytes, immunoglobulin, antibody response and macrophages phagocytic activities has been highlighted in thermal-stressed poultry birds (18, 19).

Blood glucose levels are raised by corticosteroid-stimulated gluconeogenesis. Corticosterone concentration changes have an impact on body composition, meat quality, and protein and lipid metabolism (20). Thyroid hormones (thyroxine and triiodothyronine) secreted by the thyroid gland, are essential for regulating metabolic rate. Additionally, they are indicators that depict the immune status of poultry birds during heat stress. Reduction in antibody level (21), the relative weight of lymphoid organs (spleen, thymus, bursa), and the liver have been reported in heat-stressed poultry birds compared to non-stressed birds (22, 23). High temperatures in tropical countries, particularly during the dry season, are linked to the prevalence of infectious and contagious poultry diseases like Newcastle disease and Gumboroo sickness (24).

### Metabolic responses to heat stress in poultry

Modern-day commercial broiler chickens have a rapid metabolic rate and greater heat production, thus prone to heat stress (25). Essentially, chickens’ metabolic processes are altered by high ambient temperatures, which also causes them to produce more glucose to maintain homeostasis (26). Maeda et al. (27) indicate that organisms under heat stress have a higher catabolic activity to produce energy to combat heat stress. The energy required for protein synthesis and breakdown is high: 4.5–7 mol of ATP are needed for each peptide bond created, and 1–2 mol of ATP are needed for each broken peptide link (28), suggesting that catabolic processes are the cause of the rise or fall in free amino acids that happen due to thermal challenge (29). Air sacs are crucial for gaseous exchange at high temperatures because they promote surface air circulation. Evaporation, therefore, results in heat disposal (30). It is important to note that greater panting results in higher blood pH (respiratory alkalosis) and more carbon dioxide being exhaled (31).

Poor growth performance in poultry has been ascribed to heat stress, which disrupts immune and intestinal functions, causes endocrine dysfunction, and increases oxidative stress (6, 32, 33). The hypothalamic axis is stimulated by heat stress due to an increase in adiponectin and leptin levels, resulting in a reduction in feed consumption (34). Animals under thermal stress can reduce their heat generation by minimizing their feed intake. In turn, protein deposition is adversely affected during the thermal challenge. Thermal stress influences ribosomal gene transcription and synthesis of proteins with a resultant lower deposition of protein (35). The findings of Le Bellego et al. (36) reveal that a high-protein ration elicited lower deposition of fat in animals under stress than in high-energy and fat feed.

Generally, exposing birds to adverse environmental temperatures results in a decline in thyroid activity and protein contents while increasing protein catabolism, metabolic acidosis and anaerobic glycolysis. By disrupting mitochondrial function, heat stress reduces aerobic metabolism, reduces aerobic metabolic activity, and increases glycolysis, which leads to an increase in muscular fat deposition (37). In homoeothermic birds, whose body temperature must be kept high and steady, triiodothyronine and thyroxine hormones are considered to be the primary regulators of metabolic heat generation (38). Triiodothyronine and thyroxine hormones are involved in thermogenesis in poultry species To maintain a normal body temperature. The thyroid hormone improves basal metabolism through the modification of mitochondrial function and helps skeletal muscles adapt to varying environmental conditions (39). Thyroid size and functions have been demonstrated to be impaired during thermal stress (40). Similar to the findings of Atta (41), a study conducted by Tollba and Hassan (42) found that chickens exposed to thermal challenge (38°C for 3 h daily from 35 to 40 days of age) exhibited lower plasma T3 concentrations. Additionally, the report of Mahmoud et al. (43) showed that chickens challenged with heat stress had lower levels of T3. Moreover, exposing quails to a high ambient temperature (38°C for 24 h) reduced plasma T3 compared to those under a thermo-neutral zone (44). Existing data indicate that downregulation or upregulation responses of chickens subjected to thermal challenge may be influenced by age and stress duration/level. For instance, the findings of May et al. (45) indicated that chickens exposed to 41°C did not influence thyroid hormones (T4 and T3) concentrations. However, a 1-h thermal challenge of 50°C in a 5-day-old chick increased thyroxine and triiodothyronine concentrations (46). In contrast, Tollba and Hassan (42) indicated that 3 h of exposure of chickens to 38°C reduced the blood T3 and Zaglool et al. (47) revealed that exposing broilers to thermal challenge (36°C) for 6 h daily elicited a decline in plasma T3 between weeks 4 and 6. Thermal manipulation (2-h exposure of embryos to 39.5°C on days 3, 7 and 13) during embryonic development increased the concentrations of thyroid hormones in quails (48). Assessing T3 concentrations in heat-stressed birds could be utilized as an evaluation tool since they play essential roles in raising metabolism by slowing down the rate of the oxidation of glucose and enhancing the metabolic heat generated (49).

The decline in the blood thyroid hormone during heat stress serves as an adaptive mechanism to reduce maintenance energy requirements and metabolic heat production and increase fat deposition by disabling lipolysis to escape additional heat load (50). The demand for energy increases under heat stress. The findings of Bowen and Washburn (46) have revealed that thyroid hormones from external sources had a shorter survival time under thermal challenges. Impaired metabolic changes could result in poor performance of chickens. Chronic and acute thermal challenges could lead to a decrease in the birds’ metabolism, resulting in severe complications on the performance of broilers, including decreased meat juiciness, water holding capacity, muscle pH, color, and muscle pH (40, 51).

The HPA axis is activated by thermal challenge, which also alters the neuroendocrine system’s function in poultry. Elevated plasma corticosterone levels can also impact cellular transport, proliferation, cytokine release, and antibody formation (23). The increase in corticosterone levels during stress may be related to the broilers’ impaired intestine absorptive abilities and morphology (52). The metabolic and overall immunological response of broilers to thermal challenge may be significantly impacted by an increased corticosterone concentration (53).

Recently, Yuanyuan et al. (54) indicated that broilers’ ability to resist stress and oxidative damage steadily declined as the ambient temperature rose, and heat stress was found to have a detrimental impact on these traits. The authors identified some metabolites as possible biomarkers of heat stress in broilers, including glutaric acid, neohesperidin, tartronic acid, tartaric acid and allose. The findings demonstrated that as the temperature rose from 20°C to 30°C, the concentrations of lactate dehydrogenase, cortisol, adrenocorticotropic hormone, and creatine kinase increased in broiler chickens.

### Gut health and heat stress

The gastrointestinal tract is a biological environment for the breakdown and absorption of nutrients as well as defense against diseases and toxins (8). The gut is frequently perturbed by different stressors since it is the greatest body surface exposed to the environment (55). Chickens’ gut epithelium can be impacted by heat stress (56). Stress causes the right junction-regulated paracellular barrier to become unstable and raises the permeability of the gut (57). The findings of Quinteiro-Filho et al. (58) revealed that birds subjected to thermal challenge had mild multifocal enteritis. Additionally, the gut microbiota is impacted by a wide range of host- and environment-related factors (59). In particular, thermal challenge disturbs the gut microbiota composition via decreasing the beneficial bacteria but increasing the harmful bacteria. Previous studies show that heat stress had a significant impact on gut microbial organisms by increasing the number of *Coliforms* spp., *Clostridium perfringens,* and *Escherichia coli* while decreasing levels of *Bifidobacterium* and *Lactobacillus* spp., causing dysbiosis (60, 61). A change in the gut microbiota composition results in an excess of potentially hazardous bacteria or a decrease in helpful bacteria, which can undermine the delicate microbial balance in the gut (62), resulting in gut inflammation and poor nutrient absorption (62). In contrast, a balanced microbial ecosystem (eubiosis) would improve the gut health, nutrient metabolism, growth performance, and thermotolerance of birds (63). Thus, there is a need for more studies to understand the influence of thermal challenges on gut microbiota and intestinal health.

The use of antimicrobials as a prophylactic approach to minimize the effect of stress on both gut microbiota and health in poultry is a topic of considerable interest and concern in the poultry industry. With the ongoing ban on antibiotic growth promoters, several feed additives have received growing attention in the poultry industry as natural antibiotic alternatives. These include essential oils, organic acids, symbiotics, prebiotics, probiotics, enzymes, and phytogenes such as oleoresins, botanicals, and herbs (64). Besides their effectiveness as growth promoters, prebiotics have also been shown to have a beneficial impact on the gut microbiome and immunological condition of birds during heat stress (65). Intensifying the use of natural antibiotic alternatives as growth promoters on a large scale provides safe and healthy substitutes that possess a wide range of beneficial properties such as immune-modulating effects, enhanced digestion, improved performance, improved nutrient absorption, increased absorbability, improved gut health, lesser risk of antibiotic resistance, and safe consumption of poultry products.

### Mitigation strategy to reduce heat stress in poultry

#### Environmental strategies

Timely and effective management of environmental conditions can reduce the negative effects of heat stress. The housing system is a potential stressor in production and housing management is key in combating heat stress. Depending on the environment, rearing systems and accessories present in poultry housing facilities might have a role to play in heat stress management. It has been highlighted that failure of the temperature regulation and ventilation controls in environmentally controlled housing could result in heat stress (1, 66). In open-sided buildings or non-environmentally controlled housing, poor stocking density and poor ventilation can compound heat stress problems (67–69). Fans, interior fogging, and sprinkler systems have all been employed successfully in this type of building (70). In general, both environmentally controlled and non-controlled housing can reduce heat stress through optimal ventilation and the availability of required cooling equipment. Reduced stocking density has also been shown to be one of the most efficient ways to manage heat stress. Chickens under heat stress spend less time moving around and standing still, eat less food and drink more water, stretch their wings, pant, and dustbathe (4). Some efficient management techniques include reducing the stocking density of birds to improve access to feed and water (71), and proper litter management to enhance dustbathing.

#### Thermal conditioning

Thermal conditioning (embryonic and early thermal conditioning) is a promising tool in heat stress management and has been demonstrated to enhance the ability of birds to tolerate heat during their adult life. The application of thermal conditioning within the first few days of life is capable of decreasing body temperature at an older age (72) by enhancing the development of temperature regulatory mechanisms. An important factor to note is that the consequential thermotolerance level in adult life is significantly dependent on the duration of early thermal conditioning as demonstrated by Oke et al. (73). Additionally, the findings of Meteyake et al. (74) established a positive and long-lasting effect on the survival and performance of chickens in a hot environment.

The mechanism through which early thermal conditioning enhances heat tolerance is by inhibiting the production of an uncoupled protein and by enhancing HSP70 synthesis (75, 76). Embryonic thermal manipulation has been employed as an adaptive strategy to ameliorate heat stress and enhance the adaptive capacity of birds. Although Al-Zghoul and El-Bahr (77) opined that stabilized incubation temperature during embryonic development could positively impact post-hatch thermotolerance. Loyau et al. (78) demonstrated manipulating temperature for thermal conditioning of broiler birds during embryonic development has an impact on gene expression of the pectoralis major muscle. The authors reported a more developed specific pathway involving epigenetic processes, anti-apoptotic, vascularization, stress response, and genes related to metabolism.

#### Genetic strategies

Genetics has a role to play in response to heat challenge (79). Genetic selection is a promising tool in developing heat-resistant breeds. For instance, the Naked neck gene, which is the sole dominant autosomal gene, reduces the number of feathers in the neck region of birds, allowing the neck region to disperse heat. In broilers, the naked neck gene is linked to an increase in breast muscle and body weight (80), lower body temperature (81), and heterophile-to-lymphocyte ratio during the hot season (82). Interestingly, Van Goor et al. (83) demonstrated that fine-mapping with quantitative trait loci (QTL) can enable efficient screening of heat tolerance in birds. These results suggest that it is possible to use these genes to develop chickens that can withstand thermal challenges. Some of the genetic strategies are discussed below:

#### Marker-assisted selective breeding

Molecular markers have recently been developed to locate potential candidate genes connected to heat-tolerant features for chicken bird selection to increase resistance to heat stress (84). By increasing the capacity of chickens to survive hot environments, such genetic potential can aid the poultry industry in improving the general performance of poultry (85). In order to create thermotolerant breeds using marker-assisted selection, it may be essential to identify and include the right biomarkers in breeding programs for thermal stress reactions in chickens (86). For example, HSP70 and HSP90 genes are known to exert a protective function in the body against the detrimental effects of oxidative stress and are utilized as a marker for heat stress in poultry (87–89). In both domestic and commercial hens, a silent mutation in the HSP70 coding area could serve as a marker for heat tolerance (90). According to Mahmoud et al. (91), the amount of chicken HSP70 mRNA expression in the heart and liver of young White Leghorns was strongly linked with body temperature. Selective breeding with such potential candidate genes could improve the thermotolerance of birds.

#### The naked neck gene

In chicken, the naked neck (Na), dwarf (Dw) and frizzle (F) genes are considered as candidates for temperature stress tolerance. They offer a practical, sustainable, and cost-effective solution to the heat stress challenge (92). Utilizing beneficial heat-resistant genes such as slow feathering (K), frizzle (F) and naked neck (Na) might increase heat tolerance, growth performance, and reproductive qualities in chickens (7, 93, 94).

When compared to hens with normal plumage, homozygous chickens with the naked neck gene (Na) have roughly 40% less feather coverage, and heterozygous siblings have between 20 and 30% less (93, 94). When exposed to high ambient temperatures, the reduced plumage allows them to dissipate heat (95, 96). According to studies, birds with Na performed better under heat challenge than normal-feathered birds. It has been found that this gene can tolerate harsh environmental changes, such as high temperatures (97, 98). The report of Lin et al. (7) and Rajkumar et al. (99) revealed that the Na chicken line had improved growth and immunity. The absence of feathers on the neck increases the area available for heat dissipation and discourages heat insulation, which helps birds withstand the sweltering heat (39). Galal et al. (100) discovered that in indigenous Egyptian breeds raised under heat stress, the gene enhanced thermotolerance by raising HSP70 gene expression. During the summer, the naked-necked birds’ H/L ratio and total plasma cholesterol were much lower than those of usual birds (82). Egg weights, quality, and number in laying hens with the naked neck gene improved under heat stress (93). The Na gene might be regarded as a marker gene since various genotypes can be distinguished by visual inspection after hatching based on the appearance of their feathers.

#### The frizzle gene (F)

Another gene that could be targeted for producing heat-tolerant chickens is the frizzle gene. The frizzle gene (F) is a partially dominant gene that decreases feather intensity, thereby increasing the excessive heat-dissipating ability of birds (94). According to Lin et al. (7) and Wasti et al. (33), the frizzle (F) gene causes the shape of the feather to curve, which reduces the feather’s weight and boosts heat emission from the body. The feathers in adult frizzled birds (FF and Ff) are more fragile and curled than in the typical condition (ff). With the exception of sexual maturity under heat stress, Sharifi et al. (101) found a substantial interaction between the environmental temperature and feathering genotype (FF) for all reproductive variables, including chick production, hatchability, and egg production. The authors indicated that normally feathered hens showed a clear decline in all reproductive indices at higher temperatures in comparison to frizzle-feathered hens. The findings of Haaren-Kiso et al. (102) revealed that frizzle layers outperformed normal feathered hens in a climate chamber at high temperatures. Commercializing naked-necked and frizzled birds will be beneficial to developing nations in tropical climates. Greater heat dissipation and low feather intensity are produced by the combination of the Na and F genes, especially when the Na gene is homozygous (NaNaF-) and the double heterozygous (Na/Na F/f) broiler has an additive impact (103, 104).

#### Dwarf (dw) gene

Approaches that emphasize heat tolerance and investigate the potential of indigenous chicken features such as dwarfism (dw), are important for thermoregulation (85). Homozygous males and females with the dwarf gene have lower body weights of roughly 40 and 30%, respectively and it is considered to be thermal-tolerant (7, 39, 70, 105). This might result in dwarf commercial broilers having an innate resistance to thermal challenge in harsh tropical environments. According to Merat (106), birds with the dwarf gene had body weight reductions of 33% and feed consumption reductions of 20–25%. In comparison to their normal-sized siblings, the dwarf birds showed a number of pleiotropic effects and benefits during thermal challenge, including higher resistance to disease, reduced feed consumption, improved feed efficiency, and better reproductive fitness (85, 94). However, other studies indicated no practical values (107, 108).

#### HSP 70 polymorphism

The HSP70 gene polymorphisms may help poultry produce heat-tolerant capacity. In heat-tolerant chickens, polymorphisms occur in the coding and regulatory regions of HSP70 (109). HSP70 expression is highly activated in various tissues under thermal challenge in different poultry species such as quails, turkeys, chickens, etc. (110–112). The findings of Kennedy et al. (113) established a structural polymorphism in the HSP70 gene, as shown by changes in the partial HSP70 gene, among Kenyan chicken ecotypes.

### Nutritional manipulation for heat stress alleviation in poultry

**Figure 1:**
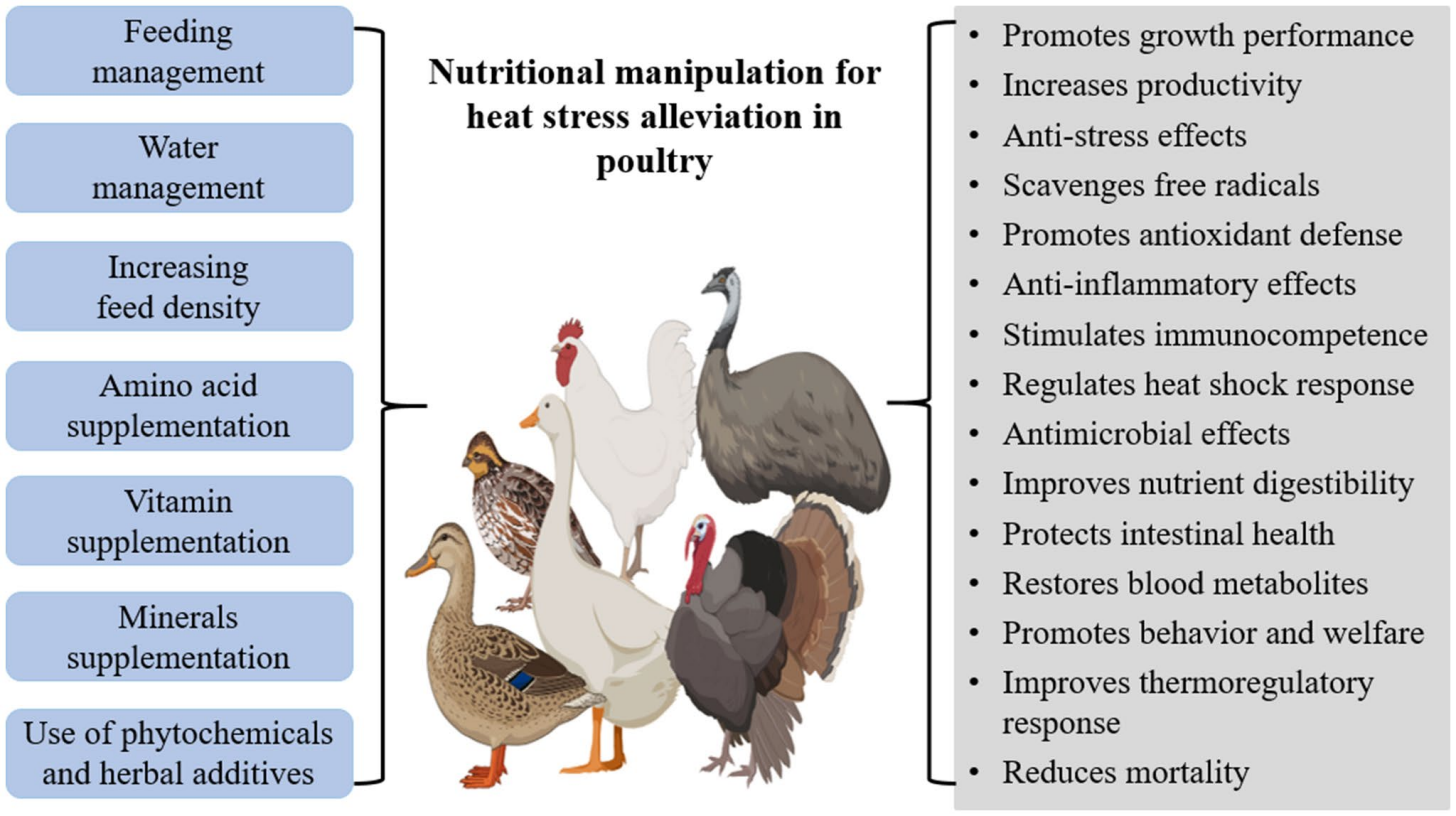
Nutritional manipulation strategies and their associated benefits for heat stress alleviation in poultry.

Nutritional manipulation is an acceptable method for the amelioration of heat stress effects in poultry and it is often applied with other management, environmental and genetic strategies (114). It involves dietary manipulations such as elevation of feed density and dietary energy, supplementation with feed additives, nutritious compounds, phytochemicals, bioactive components, and other nutraceuticals which would offer beneficial biological effects to alleviate heat stress effects (115, 116). These substances may act as anti-stressors, growth modulators, antioxidants, anti-inflammatories, immunomodulators, gut modifiers, etc. as shown in Figure 1.

## Feeding management

Typically, feed consumption and nutrient intake decrease during heat stress in poultry, negatively affecting the performance and productivity of birds. Therefore, modifying the nutrient composition to improve the feed intake of chickens is an important consideration during heat stress. Feeding strategies that would maximize feed intake, minimize heat load, and alleviate the negative effects of heat stress in poultry are highly desirable for the nutritional management of birds under high temperatures (117). Syafwan et al. (118) reported on several feeding strategies that may help reduce heat load in poultry such as restricted feeding during the hot periods to minimize heat load, choice feeding of protein or energy-rich feed ingredients, supplying different particle size feeds or structures that will slow the digestion process and feeding wet diets to simultaneously promote water intake.

Feed withdrawal, which is usually practiced for 6 to 8 h per day during the hottest periods of the day can potentially minimize heat increment and the adverse effects of heat stress in poultry. Feed withdrawal limits heat load accretion occurring from metabolic heat generated during the processes of feed digestion, absorption, assimilation, and excretion. Thus, it is important to limit feeding to the cool hours of the day or preferably feed during the morning, evening, or nighttime to minimize the impacts of heat stress. The heat produced from feed consumption is high, such that feeding at 06:00 h will cause a peak heat load between 09:00 to 11:00 h. Therefore, feeding toward mid-day would accrue negative additive effects since the heat generated from feed utilization would coincide with the hottest part of the day, especially during the summer period in the tropical and subtropical regions (119). With feed withdrawal during the day, the temporary feed restriction employed before heat exposure enhances thermal resistance, reduces heat production, limits the increment of body temperature, and minimizes mortality of heat-stressed poultry.

The practice of wet feeding is considerably beneficial compared to feeding conventional dry mash/pellet feed since the birds may fail to consume sufficient quantities of the dried feed under heat-stress conditions. During the summer season, laying hens given wet feed had higher yolk index, shell weight, yolk percentage, moisture percentage, and feed conversion efficiency (120, 121), thus improving the laying performance and productivity of hens. The practice of supplementing 1 part water to 1 part feed (50: 50) with drinking water was recommended for finishing broilers raised in the tropics, since wetting the feed provides an additional advantage of supplying both feed and water to heat-stressed birds, in turn improving the feed intake, dry matter intake, weight gain, carcass yield, feed conversion ratio, and growth potential of birds (122).

## Water management

Under high temperatures, evaporation serves as a medium for heat loss in poultry. This mainly occurs during panting activity which helps dissipate internal heat through water evaporation from the moist lining of the respiratory tract during the breathing process (56). Chickens also lose heat from the comb, wattles, and skin of birds through vasodilation since the blood vessels become wider allowing for increased blood circulation to the skin surface, thus helping the animal lose heat to its environment (123). A practical nutritional technique to minimize heat production, promote heat loss and enhance tolerance under high temperatures is to provide water, either for drinking or mixed with the diet for consumption. The provision of wet feed and cold water is important in supporting the metabolism, homeostasis, and physiological responses of birds. It will also minimize water excretion in fecal droppings, consequently increasing the water available for evaporation during panting (119).

The supply of cold water as against tap water influenced various physiological variables since it decreased the tonic immobility, body temperature, and blood levels of cholesterol, AST, and ALT, whereas, it increased the globulin, glucose, and total antioxidant capacity of the birds (124). Importantly, the provision of wet feed and cold water during high summer temperatures improved the body weight and body weight gain of ducks (124). Supplying cold water with afternoon feed withdrawal was found to decrease body temperature and tonic immobility, whereas, it improved the production performance, blood composition, and the total antioxidant capacity of Muscovy ducklings during summer heat (125). Thus, simultaneously using feeding systems such as feed withdrawal or wet feeding and cold water supply is recommended to alleviate heat stress in poultry. Additionally, the oral provision of some nutrient supplements is efficacious in promoting rehydration and alleviating heat stress in poultry. This would enhance water utilization and increase its retention rate, thus helping birds cope with heat stress (126).

## Increasing the energy content of the diet

Supplementing with fats and oil to increase the energy content of feeds is another method for the nutritional manipulation of heat-stressed poultry. Fats and oils have high energy value, lower heat increment, and are crucial to the absorption of fat-soluble vitamins, nutrient digestibility, and utilization. In heat-stressed animals, the dietary inclusion of fats and oils promotes feed intake, enhances productivity, and minimizes heat load (127). The improvement in growth performance occurs due to higher energy/fat intake since fats and oil enhance feed palatability and allow for reduced heat increment relative to protein and carbohydrates rich diets (128). Increasing the dietary metabolizable energy up to 3,300 kcal/kg and adding fat up to 5% dietary fat improved the performance, nutrient digestibility, and carcass traits of heat-stressed broiler chickens (129). In another study, broiler chickens supplemented with up to 8% dietary vegetable oils showed improved production performance, meat quality traits, blood hematological and biochemical indices, antioxidant properties, and immune responses, thus exhibiting heat stress tolerance (130).

Ultimately, increasing the fat content of the diet contributes to minimizing heat production since fat metabolism requires greater efficiency and produces lesser heat increment compared to protein or carbohydrate metabolism (116). Compared to only increasing the protein concentration, an increase in protein levels (from 19 to 22%) with increasing energy concentration (from 13.18 to 13.81 MJ/kg) using oil supplementation improved the body weight gain protein intake and European performance index in heat-stressed broilers (128). Hence, since heat stress decreases feed consumption, nutrient intake, and metabolizable energy, it is recommended that the dietary inclusion of fats and oils would help increase the metabolizable energy intake and reduce heat increment in poultry (128).

## Supplementation with amino acids

Exposure to heat stress negatively affects the availability, transport, intestinal uptake, absorption, and utilization of amino acids (116, 131). Thus, the utilization of feed-grade amino acids to supplement poultry diets has significantly increased in recent times. The provision of amino acids to meet the nutritional needs of poultry supports the productivity, intestinal health, immune response, behavior, and welfare of birds (132). Alongside this, the maintenance of amino acid balance and the supply of adequate amounts of amino acids, especially for limiting amino acids such as arginine and lysine is highly beneficial to minimizing heat stress effects (69). Sulfur-containing amino acids such as methionine and cysteine are important in poultry nutrition. Methionine supplementation decreased muscle oxidation and improved the tissue antioxidant status in heat-stressed broilers (133). In another study, supplementation with sulfur amino acids alleviated chronic heat stress via increasing antioxidant production and protecting the intestinal permeability of broiler chickens (134, 135). Glycine, a conditionally indispensable amino acid in poultry is also essential in enhancing production performance, and alleviating oxidative stress and intestinal dysfunction in heat-stressed birds (131, 136).

Additionally, some non-essential amino acids and derivatives including taurine, L-theanine, betaine, and L-citrulline have emerged as functional nutraceuticals for heat stress alleviation in poultry. These bioactive compounds possess potent biological properties that enable them to function as anti-stressors, antioxidants, anti-inflammatories, immunomodulators, and gut stimulants when supplied to heat-stress poultry (131). Importantly, the supplementation of low–crude protein diets with limiting amino acids has become a useful nutritional strategy to address the negative impacts of heat stress and minimize the environmental impact of poultry production (137). The use of reduced crude protein diets is a useful nutritional technique for feeding heat-stressed birds since it minimizes the use of high-protein feed ingredients, lowers nitrogen excretion, changes manure composition, reduces gaseous emissions, and consequently, decreases the carbon footprint, and consequently lower environmental impact from feed production (138).

## Supplementation with vitamins

Vitamins supplementation is a nutritional approach that is highly useful during heat stress in poultry. Importantly, most of these vitamins play crucial roles as anti-stressors, antioxidants, immunomodulators, anti-inflammatories, gut protectants, and growth promoters. It is known that chickens do not undergo significant endogenous synthesis of vitamins to meet nutritional needs, especially under heat stress conditions, since high ambient temperature may decrease their biosynthesis and retention, and alter metabolic functions (139). As such, the exogenous provision of one or a combination of vitamins would prove useful against the adverse effects of heat stress in poultry. Commonly, Vitamins A, B, D, E, and C are utilized to promote immunocompetence and antioxidant response during heat stress in poultry (140, 141). For instance, Vitamin A supplementation promoted the feed intake, laying rate, and egg weight of laying hens and further increased the proportion of peripheral T lymphocytes, thus improving both the laying performance and immune function of heat-stressed hens (142). Vitamin C is an important metabolite that acts as a reducing agent and an electron donor, thus serving as a potent natural antioxidant. Vitamin C supplementation in feed or water is well known to alleviate the adverse effects of heat stress in poultry. Vitamin C supplementation at ~250 mg/kg feed has been optimized to improve the production performance, nutrient digestibility, immune responses, and antioxidant capacity in heat-stressed poultry (139). Vitamin E is another important antioxidant that is present in the body’s system. It elicits protective effects during heat stress and alleviates the negative impacts on growth performance, productivity, nutrient digestibility, immunity, and antioxidant profile in poultry birds (143, 144). Supplemental doses from 200 to 500 mg/kg body weight have been found efficacious in mitigating heat stress effects in poultry.

The combination of vitamins is considered more efficient than the use of an individual vitamin in alleviating heat stress in poultry. It has been demonstrated that supplementing the combination of vitamins C and E would prove more beneficial than their sole supply due to their synergism and antioxidant properties in combating heat stress (128). Vitamins C and E combination improved the egg quality traits of heat-stressed hens (145), as well as the feed efficiency, growth performance, and immune responses in heat-stressed chickens (146). Alongside this, vitamins have often been supplied in combination with minerals or other substances to elicit synergistic effects. Dietary inclusion with a multi-complex of vitamin E, vitamin C, and Selenium did not cause any negative effects on the carcass traits, oxidative capacity, and meat quality of heat-stressed birds (147). Similarly, dietary incorporation of vitamin E and organic selenium reduced mortality and improved the growth performance and carcass characteristics of heat-stressed broilers (148). In a follow-up study, vitamin E and selenium were found to exert beneficial synergistic effects that ameliorated heat stress effects via improving the antioxidant capacity, modifying the ileal microbiota, and regulating the mRNA expression of several cytokines (149).

## Supplementation with minerals

Decreased feed intake results in an unmet fulfillment of the mineral requirements of heat-stress birds. Minerals support various cellular and biological functions, sustain growth and productivity, improve nutrient utilization, boost immunity, and attenuate oxidative stress in heat-stressed poultry (150). Exposure to heat stress causes respiratory alkalosis which creates a negative mineral balance and increase losses in sodium and potassium ions during excretion in chickens. Increased mineral excretion is a negative consequence of heat stress in poultry that leads to acid–base imbalance, which can be alleviated by supplementing appropriate mineral elements at different stages of production (145). For instance, the addition of potassium chloride to the drinking water of heat-stressed chickens significantly improved the body weight gain, decreased body temperature, and reduced the blood pH of heat-stressed chickens, thus improving the physiological adaptation of birds to the stressor (151).

Zinc is a trace element that serves as a cofactor for several enzymes and its supplementation is essential since it cannot be stored within the body (152). Zinc elicits protective effects during heat stress since it can eliminate reactive oxygen species, enhance antioxidant ability, and attenuate heat shock response. Zinc supplementation to turkey breeders during the hot summer period increased egg laying and the occurrence of behavioral activities such as dustbathing and feather cleaning (153). In another study, zinc addition decreased the plasma corticosterone levels as a biomarker of stress induction, and it increased the egg production and body weight of turkey breeders during summer heat (154). Another element, selenium is highly beneficial during heat stress due to its potent antioxidant and immuno-enhancing properties. Dietary supplementation with selenium improves the production performance, egg production, egg quality, immune system, and antioxidant status of heat-stressed poultry (155). Manganese is also demonstrated to protect against heat stress by promoting antioxidants expression and attenuating heat shock responses (156). Chromium is another essential trace element that is increasingly mobilized from body tissues and excreted during heat stress, thus increasing its nutritional requirement (157). Chromium supplementation is beneficial for heat stress alleviation by increasing the production performance, carcass traits, nutrient digestibility, immune response, and oxidative stability of poultry (158). Dietary chromium supplementation improved the feed intake, hemoglobin, packed cell volume, and blood biochemical indices of Japanese quail under heat stress (159). Thus, along with the above-mentioned minerals, several trace elements including calcium, phosphorus, copper, iron, sodium, potassium, magnesium, and iodine have been studied and found beneficial in mitigating heat stress in poultry (150).

## Use of phytochemicals and herbal additives

The use of plant extracts has gained precedence as a nutritional strategy to ameliorate thermal stress in poultry owing to their ease of availability, potency, and numerous biological effects (160–168). In recent times, several plant-based and alternative substances with bioactive properties have been utilized as feed additives and nutritional modifiers for heat stress mitigation in poultry. A recent bibliometric study unveiled that several substances including flavonoids, probiotic mix, curcumin, resveratrol, essential oils and various plant extracts have emerged as beneficial dietary supplements to alleviate the detrimental effects of heat stress in poultry (169). Bioactive agents including resveratrol, curcumin, and quercetin were reported to activate vitagenes and effectively regulate the antioxidant defense system, especially the nuclear factor-erythroid 2-related factor 2 (nrf2) signaling pathway to attenuate heat stress-induced oxidative stress in poultry (170). In line with this, dietary resveratrol supplementation was found to improve growth performance, alleviated liver injury, and enhanced antioxidant capacity by increasing the activities of SOD, GPX, and the Nrf2-Keap1 signaling pathway in broiler chickens (171).

The thermoregulatory potentials of some herbs and plant products including ginger, turmeric, sweet wormwood, hot red pepper, thyme, rosemary, moringa, licorice, cinnamon, ginkgo, and resveratrol have been extensively reviewed in poultry. Dietary supplementation of these herbs is efficacious in ameliorating the negative effects of heat stress in poultry via enhancing the production performance, scavenging free radicals, promoting the antioxidant defense system, stimulating the immune system, regulating heat shock response, exerting antimicrobial effects, decreasing corticosterone release, improvements in nutrient digestibility, protecting intestinal health, regulating blood biochemical properties, influencing behavioral patterns and significantly reducing mortality of birds (115). In addition, polyphenols, often derivable from plants, can scavenge free radicals, decrease lipid peroxidation, modulate antioxidant enzyme activities, and attenuate oxidative stress thus providing a sustainable nutritional strategy for heat stress mitigation in poultry (168, 172, 173).

## Conclusion and future perspectives

A multifaceted strategy for managing heat stress should be adopted in the poultry industry. Future trends in the poultry farming industry should be poised to change how heat stress is handled in chickens. Precision climate control, utilizing cutting-edge sensors and automated systems to dynamically adjust temperature, humidity, and ventilation within poultry houses, is needed to take center stage with the growing environmental temperatures. Targeted breeding techniques are needed to generate heat-resistant poultry breeds as a result of genetic developments, and specialized diets and nutritional supplements rich in nutrients that regulate body temperature are crucial instruments for boosting birds’ resistance to heat stress. Innovative cooling techniques like evaporative cooling and radiant surfaces should also be explored. Furthermore, attention should focus on behavioral monitoring using AI-powered sensors to detect heat stress and the use of prediction algorithms to foresee heat stress and enable prompt responses. The genetic selection of heat-tolerant poultry breeds, precise climate control systems, creative cooling methods, and specialized nutritional interventions should also be fine-tuned to mitigate the influence of thermal challenges on poultry production.

## Author contributions

OMO: Conceptualization, Data curation, Formal analysis, Funding acquisition, Investigation, Methodology, Project administration, Resources, Software, Supervision, Validation, Visualization, Writing – original draft, Writing – review & editing. VU: Conceptualization, Formal analysis, Funding acquisition, Investigation, Methodology, Project administration, Resources, Software, Supervision, Validation, Visualization, Writing – original draft, Writing – review & editing. OO: Conceptualization, Formal analysis, Funding acquisition, Investigation, Methodology, Project administration, Resources, Software, Validation, Visualization, Writing – original draft, Writing – review & editing, Data curation. KT: Conceptualization, Methodology, Project administration, Resources, Validation, Writing – review & editing, Supervision. OEO: Conceptualization, Methodology, Project administration, Resources, Supervision, Validation, Writing – review & editing, Data curation, Formal analysis, Funding acquisition, Investigation, Software, Visualization, Writing – original draft.
